# EGb761 protects motoneurons against avulsion-induced oxidative stress in rats

**DOI:** 10.1186/1749-7221-5-12

**Published:** 2010-05-24

**Authors:** Xiao Cheng, Fo-Lin Liu, Jun Zhang, Lin-Lin Wang, Fang-lan Li, Shu Liu, Li-Hua Zhou

**Affiliations:** 1 Department of Anatomy, Zhong Shan School of Medicine; Sun Yat-Sen University, No. 74 Zhong shan Road 2, Guangzhou 510080, PR China; 2 Department of Obesterics, the Third Affiliated Hospital; Sun Yat-Sen University, No. 74 Zhong shan Road 2, Guangzhou 510080, PR China

## Abstract

**Background:**

Root avulsion of the brachial plexus causes an oxidative stress reaction in the spinal cord and induces dramatic spinal motoneuron death, while EGb761 is a natural free radical cleaning agent. This study was designed to investigate the protective effects of intraperitoneally injected EGb761 against neural damage following brachial root avulsion.

**Methods:**

The effect of EGb761 on avulsion-induced motoneuron injury was studied in 26 total groups of (n) rats, treated as follows. Animals in singular number groups received EGb761(50 mg/kg.d) and those in complex number groups received normal saline solution (i.p.), serving as controls. Groups 1-8 were used for the determination of nitric oxide (NO) levels in the serum and injured spinal cord at the 5 d, 2 w, 4 w, and 6 w time points. Groups 9-16 were used for determination of constitutive nitric oxide synthase (cNOS) and inducible nitric oxide synthase (iNOS) levels in injured spinal cord at the 5 d, 2 w, 4 w, and 6 w time points. Groups 17-26 were used for determination of the number of neuronal nitric oxide synthase (nNOS)-positive and surviving motoneurons in injured C7 ventral horn at the 5 d, 2 w, 4 w, 6 w and 8 w time points.

**Results:**

Compared to control groups, the EGb761 treatment group not only had significant decreased levels of NO in serum at 2 w and 6 w after avulsion, but also had reduced levels of NO specifically in the spinal cord at 2 w, 4 w and 6 w. The cNOS activity in the spinal cord was also significant decreased at 2 w and 4 w, while the iNOS activity in injured C6-T1 spinal segments was reduced at 2 w, 4 w and 6 w. All together, the percentages of NADPH-d positive motoneurons in an injured C7 segment were down-regulated and the number of surviving motoneurons in injured C7 ventral horn was increased at 2 w, 4 w, 6 w and 8 w in treated versus untreated animals.

**Conclusions:**

Intraperitoneal administration of EGb761 after root avulsion of the brachial plexus exerted protective effects by decreasing the level of NO in spinal cord and serum and the activity of cNOS and iNOS, easing the delayed motoneurons death. EGb761 should be considered in the treatment of brachial plexus nerve injuries.

## Introduction

Brachial plexus injuries in adults are commonly caused by auto or motorcycle accidents. The treatment of this type of injury consists of nerve repair and nerve grafting for extraforaminal nerve root or trunk injury, and of neurotization or nerve transfer for nerve roots avulsion; however, the outcome of brachial plexus reconstruction and the restoration of shoulder and elbow function are often poor in spite of the sophistication of the various methods used[1,2]. The death of a major proportion of the innervating neuronal pool is likely to be the most fundamental neurobiological barrier to functional restitution because survival is an essential prerequisite for regeneration[3]. Currently the primary aim of management of root avulsion of the brachial plexus is motor recovery. However, 80-90% of motoneurons have been shown to die after avulsion[4,5], complicating this goal. Immediate repair or nerve grafting offers some degree of protection to the motoneurons but is clinically limited, so there remains a need for medical approaches to maintain the viability of the injured motoneurons.

The Ginkgo biloba extract EGb761 is a standardized mixture of active substances obtained from green leaves of the Ginkgo biloba tree, composed of 24% flavonoid glycosides and 6% terpenoids[6,7]. EGb761 has been reported to be a potent free radical scavenger and many studies have demonstrated that the compound affects hemodynamics, metabolism, and hemorrheology. Additionally, EGb761 has antioxidant properties and transmitter/receptor effects in the brain, spinal cord, peripheral nervous system, retina, vestibulocochlear apparatus and cardiovascular system[8-10]. The mechanism of EGb761 action in the central nervous system (CNS) is relatively well-studied, and the main effect it exerts seems to be related to its antioxidant properties. Recently, in vitro studies have shown that EGb761 has a protective effect against neuronal apoptotic death[11,12] and an inhibitory effect on the expression of inducible nitric oxide synthase (iNOS) and nitric oxide (NO) production [13]. Animal experiments have also shown that EGb761 can prevent neuronal damage after brain ischemia through the inhibition of NOS [14]. However, its potential effect in patients suffering from spinal cord injury (SCI) is still unknown. In our previous studies, de novo expression of neuronal NOS (nNOS) was observed in injured motoneurons, and the time course and density of nNOS expression both correlated well with the severity of motoneuron death following brachial root avulsion, in which the oxidant peroxynitrite played an important role [15,16]. This raises the question of whether EGb761 has a similar neuroprotective effect on avulsion-injured motoneurons. In the present study, we used EGb761 to treat rats immediately after avulsion injury. The effect of EGb761 was estimated according to the survival of injured motoneurons. The investigation of the protective mechanism of EGb761 was focused on the production of NO, and the activity of both nNOS and iNOS. Our present study found that EGb761 protects motoneurons against avulsion injury and that this neuroprotective effect was related to the reduction of both NO and NOS in the injured spinal cord.

## Materials and methods

### Animals and Surgery

Adult male Sprague-Dawley rats (250-280 g) were obtained from the Laboratory Animal Center of Sun Yat-sen University, and all procedures were approved by the Committee for the Use of Live Animals in Teaching and Research at Sun Yat-sen University. All rats had free access to standard rat chow and tap water. Rats were fed with standard rat diet routinely, but were deprived of food for 12 h before the first operation. All rats in the present study received root avulsion surgery. Spinal root avulsion surgery followed the procedures described in our previous publications [4,17,18]. Briefly, the rats were anesthetized with intramuscular injections of ketamine (80 mg/kg) and xylazine (8 mg/kg), and all nerve roots, including C5, C6, C7, C8 and T1 of the right brachial plexus, were separated under an Olympus surgical microscope. Extra-vertebral avulsion of the ventral and dorsal roots was carried out on C5, C6, C7, C8, and T1 by pulling the nerve root out with microhemostatic forceps. The avulsed ventral and dorsal roots together with the dorsal root ganglia were cut away from the distal ends of the spinal nerves and examined under the microscope to confirm the success of the surgery. All surgical instruments were appropriate for the size of each animal. The skin was then sutured, and long-acting penicillin (3,000,000 units, sc) was given after the surgery. The rats were allowed to recover until awake and returned to their cages.

### EGb761 treatment

Rats received daily intraperitoneal (i.p.) injections of vehicle (saline) or EGb761 (50 mg/kg body weight). EGb761 treatment was started immediately after the root avulsion surgery. EGb761 was provided by Schwabe Pharmaceuticals (Karlsruhe, Germany). The extract is well-characterized[19] and is being used in ongoing clinical trials[20]. EGb761 was dissolved in physiological saline and the pH adjusted to 7.4. The survival time points were 5 days (5 d), 2 weeks (2 w), 4 weeks (4 w), 6 weeks (6 w) and 8 weeks (8 w) post-injury. The effect of EGb761 on avulsion-induced motoneuron injury was studied in the following experimental groups. Animals in Groups 1, 3, 5, 7, 9, 11, 13, 15, 17, 19, 21, 23 and 25 received EGb761 while those in Groups 2, 4, 6, 8, 10, 12, 14, 16, 18, 20, 22, 24 and 26 received normal saline solution (i.p.), serving as controls. Groups 1-8 were used for determination of NO levels in the serum and injured spinal cord at the 5 d, 2 w, 4 w, and 6 w time points. Groups 9-16 were used for determination of cNOS and iNOS levels in injured spinal cord at the 5 d, 2 w, 4 w, and 6 w time points. Groups 17-26 were used for determination of the number of nNOS-positive and survival motoneurons in injured C7 ventral horn at the 5 d, 2 w, 4 w, 6 w and 8 w time points. Each treatment group included six to eight rats.

### Determination of NO level

24 h after the last EGb761 or saline administration, the rats were anesthetized with a lethal dose of 10% Chloral Hydrate and 1 ml of blood was taken from the caudal vein, after which they were sacrificed by cervical dislocation. Using dorsal laminectomy, the spinal segments from C5 to T1 were identified and removed. The level of NO in spinal cord and serum was determined using a NO kit (Nanjing Jiancheng Institute of Biology and Engineering, Nanjing, China). Briefly, the method involved measuring the levels of NO metabolites (nitrite and nitrate), which are more stable than NO. We thus estimated the level of NO in the sample by determining total nitrate and nitrite concentration. The rationale for this method is based on the fact that nitrate reductase catalyzes the enzymatic conversion of nitrate to nitrite and determines total nitric oxide concentration. This step was followed by the colorimetric measurement of nitrite as an azo dye product of the Griess reaction. A two-step diazotization reaction occurs during the Griess reaction, wherein acidified nitrite produces a nitrosating agent that reacts with sulphanilic acid to produce the diazoniumion. This product is then coupled with N-(1-naphthyl) ethylenediamine to form the chromophoric azo-derivative, which has a peak absorbance of 550 nm. The NO level in spinal cord was expressed as μmol/g of spinal cord protein. The NO level in serum was expressed as μmol/L of serum[21].

### Determination of constitutive NOS (cNOS)

Rats were killed by cervical dislocation 24 h after the last EGb761 or saline administration. Using dorsal laminectomy, the spinal segments from C5 to T1 were identified and removed[15,22,23]. Inducible NOS (iNOS) activity and total NOS activity in spinal cord were measured with a NOS kit (Nanjing Jiancheng Institute of Biology and Engineering, Nanjing, China), which assessed activity by measuring the conversion of L-[^14^C]-arginine to L-[^14^C]-citrulline [24]. The total NOS activity was determined by incubating samples (50 μL) for 15 min at 37°C in a reaction mixture containing buffer solution and 20 μM nicotinamide adenine dinucleotide phosphate(β-NADPH), 1 mM CaCl_2_, 50 μM tetrahydrobiopterin (BH4) and 1 μCi/ml L-[^14^C]-arginine. Inducible NOS (iNOS) activity was measured by omitting calcium and adding 1 mM EDTA to the reaction mixture (50 μL) for 60 min at 37°C. The reaction was stopped by the addition of 1 ml of ice-chilled buffer containing 30 mM HEPES and 3 mM EDTA (pH 5.5), after which the reaction mix was applied to Dowex AG50W-X8 columns to remove L-[^14^C]-arginine. Columns were eluted two times with 0.5 ml of distilled water and L-[^14^C]-citrulline was quantified using a liquid scintillation spectrophotometer. cNOS activity was computed by subtracting iNOS activity from total NOS activity. One unit (U) of total NOS activity was defined as picomoles of L-[^14^C]-citrulline produced per minute per microgram protein/milliliter. The activity of cNOS in spinal cord was expressed as U/mg of spinal cord protein.

### NADPH-d histochemistry plus neutral red

At the end of each survival time (5 d, 2 w, 4 w, 6 w, 8 w), rats were anesthetized with a lethal dose of 10% Chloral Hydrate and perfused transcardially with saline, followed by 4% paraformaldehyde in 0.1 M PB (pH 7.4). After perfusion, the vertebral column was dissected, and the spinal cord was removed. The C7 spinal segment was defined as the region between the uppermost root and lowermost root of the C7 nerve of the contralateral spinal cord. The C7 segment of the spinal cord of each animal was removed, fixed by immersion in fresh fixative overnight and cryoprotected in 30% (v/v) phosphate-buffered sucrose overnight. Frozen transverse sections (40 μm) were cut and collected in 0.01 M PB. Every third section from each animal was used for NADPH-d histochemistry plus neutral red counterstaining. We have previously shown that NADPH-d staining recognizes NOS-containing neurons under normal conditions, and that NADPH-d labels the same population of lesioned motoneurons as both NOS-ICC and NOS in situ hybridization[4,23]. Neuronal NOS-containing neurons were stained with NADPH-diaphorase (NADPH-d) following our previous studies [15,23]. Briefly, sections were incubated at 37°C for 1 h in 10 ml of 0.1 M Tris-HCL(PH 8.0) containing 0.2% Triton X100, 10 mg NADPH (Sigma), 2.5 mg nitro-blue tetrazolium (NBT) at 37°C for 1 h and then washed with 0.1 M PB three times. The stained sections were mounted onto slides and counterstained with 1% neutral red (Sigma). These sections were used to count the numbers of nNOS-positive and surviving motoneurons.

### Counting of motoneurons

Approximately thirty cross sections (of 40 μm thickness a piece) of the C7 spinal segment could be obtained from each animal, and every third section was used for NADPH-d histochemistry plus neutral red counterstaining. In total, ten light microscopic images of the C7 ventral horn of these sections in each animal were captured (20 × and 40 × lens) with a Lucida camera attached to a Leica DFC350FX/DMIRB microscope. Data quantification and analysis were performed by two independent persons, both of whom were blinded to the treatment groups and the previous studies[4,15,23]. In NADPH-d plus neutral red-stained sections, a motoneuron with a visible nucleus in the neutral red stain was counted as a surviving cell. The number of surviving motoneurons was quantified on both the intact side and the lesioned side of the C7 section. The number of surviving motoneurons on the contralateral intact side was set as 100%. The surviving motoneruons on the lesioned side, including both the NADPH-d positive and the NADPH-d-negative but neutral red-stained motoneurons, were then counted. The number of surviving motoneurons ipsilaterally was expressed as a percentage of the number of surviving motoneurons contralaterally in the same C7 section[22,25]. The number of ipsilateral nNOS-positive motoneurons, represented by only the NADPH-d reactive motoneurons, was expressed as a percentage of the number of surviving motoneurons on the contralateral side of the same C7 section. The number of nNOS-positive or surviving motoneurons of each animal was expressed as the mean of the nNOS-positive or surviving motoneurons in the 10 serial C7 sections[15,23,25].

### Statistical analysis

The statistical calculations and data handling were performed using SPSS version 16.0. All variables were expressed as medians, mean ± standard deviation (X ± SE) with the range. A one-way ANOVA was applied to detect differences among groups followed by Tukey-Kramer multiple comparison tests. Differences were considered significant at p values < 0.05.

## Results

### Effect of EGb761 on NO levels in the serum and injured spinal cord

**Figure 1:**
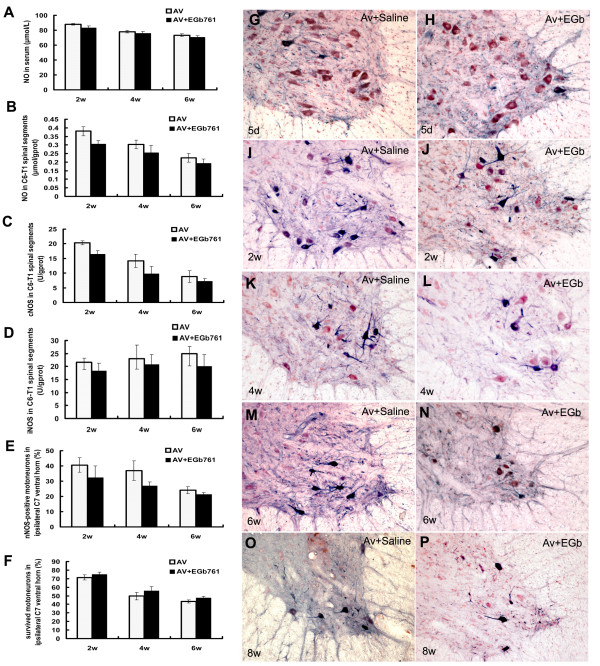
**EGb761 protects motoneurons against avulsion-induced injury in rats**. EGb761 protects motoneurons against avulsion-induced injury in rats. The neuroprotective effect of EGb761 against avulsion injury is associated with reductions in nitric oxide production and NOS expressions in the injured spinal cord. Compared to saline controls, EGb761 reduced nitric oxide levels in the serum at 2 w and 6 w, but not at 5 d and 4 w (Fig. A) and reduced nitric oxide levels in injured C6-T1 spinal segments at the 2 w, 4 w and 6 w time points (Fig. B). EGb761 down-regulated cNOS levels (Fig. C), iNOS (Fig. D), nNOS levels in injured C6-T1 spinal segments (Fig. E,F), nNOS expressions in ipsilateral ventral horn motoneurons, and the death of injured motoneurons in ipsilateral ventral horn (Fig. H,J,L,N,P) induced by C5-T1 root avulsion injury. *P < 0.01 compared with the 'avulsion+saline (Av)' group at the same time point in graphic presentations of Fig. G,I,K,M,O. Cross-sections of rat C7 segments from rats that underwent root avulsion and were injected with saline (Fig G,I,K,M,O) or EGb761 (Fig. H,J,L,N,P). Panels (G) and (H) were from rats surviving for 5 d after root avulsion. Panels (I) and (J) were from rats surviving for 2 w after root avulsion. Panels (K) and (L) were from rats surviving for 4 w after root avulsion. Panels (M) and (N) were from rats surviving for 6 w after root avulsion. Panels (O) and (P) were from rats surviving for 8 w after root avulsion. NADPH-d plus neutral red stain ×10 in Fig G-P.

Following spinal root avulsion, the levels of NO in the serum and spinal cord increased, reaching a maximum at 2 w and then gradually descending until 6 w. The neuroprotective effect of EGb761 against avulsion injury was closely related to a reduction in nitric oxide production in the serum and injured spinal cord. In serum, EGb761 reduced nitric oxide levels at 2 w and 6 w but not at 4 w (Fig. 1A) compared to saline controls, while NO levels in injured C6-T1 spinal segments of treated animals were reduced at every time point (Fig. 1B).

### Treatment with EGb761 regulated cNOS and iNOS activity in injured C6-T1 spinal segments

Root avulsion also resulted in a change in cNOS and iNOS activity in injured C6-T1 spinal segments. The activity of cNOS gradually increased after spinal root avulsion, reaching a peak at 2 w, and then descended gradually until 6 w. Meanwhile, the activity of iNOS increased gradually from the 2 w to 6 w. The activity of cNOS and iNOS were down-regulated in animals that had been administered EGb761. Quantitative analysis showed there were significant differences between the EGb761 treated group and the saline control group in the activity of cNOS in injured C6-T1 spinal segments at 2 w and 4 w but not 6 w (Fig. 1C), while iNOS activity in injured C6-T1 spinal segments showed significant differences at each time point (Fig. 1D).

### Expression of nNOS in ipsilateral ventral horn motoneurons after EGb761 treatment

There is no expression of nNOS in the undamaged ventral horn motoneurons of the spinal cord, but nNOS can be induced in motoneurons on the lesioned side following root avulsion. The number of nNOS positive motoneurons in the ipsilateral ventral horn increased rapidly to a peak at 2 w, and then decreased gradually at 4 w and 6 w, confirming our previous studies[4]. Following spinal root avulsion, nNOS labeling was widely distributed in almost every injured motoneuron and was evident in the somatic cytoplasm and dendrites (Fig. 1I, K, M), In EGb761 treated rats, expression of nNOS was significantly down-regulated. Fewer nNOS positive neurons, exhibiting weak staining in the soma, were observed on the lesioned side (Fig. 1J, L, N.) compared to the saline-treated controls. Quantitative analysis of NADPH-d-stained slides showed that the differences between the EGb761 and saline-treated control group were significant (P < 0.001) at every time point (Fig. 1E). Morphologically, many surviving motoneurons were NADPH-d-positive by histochemistry in the saline control group after injury. However, very few NADPH-d-positive motoneurons were observed at the same time point post-injury in the EGb761-treated group, and most of the remaining motoneurons were NADPH-d-negative.

### Survival of injured motoneurons after EGb761 treatment

The loss of motoneurons in the C7 spinal segments following avulsion was apparent at the end of 2 w, and was accompanied by the rapid appearance of nNOS positive motoneurons. The loss of motoneurons sharply increased at the 4 w and 6 w time points(Fig 1K-N), and the ipsilateral ventral horn showed signs of atrophy (Fig 1O), confirming our previous conclusions[4]. Quantitative analysis showed that the number of surviving motoneurons in the EGb761 treated group was higher than in the saline-treated group (Fig. 1F), and statistical analysis showed that the differences between the EGb761 and saline treated groups were significant at 2 w, 4 w and 6 w (P < 0.05). Morphologically, many of the remaining motoneurons were NADPH-d-positive in the saline control group by 6 w post-injury, while fewer NADPH-d-positive motoneurons were found at this time point in the EGb761 treated group (Fig. 1M-N).

## Discussion

The present study demonstrated that avulsion-induced motoneuron death was related to changes in nitric oxide production and NOS activity in injured spinal segments. Furthermore, we found that EGb761 prevented death of motoneurons by suppressing both iNOS and nNOS activity, thus reducing NO production in the injured spinal cord.

Extracts of Ginkgo biloba, such as EGb761, are commonly used to increase blood circulation and to protect the lipid portion of cellular membranes against damage induced by free radicals [26]. EGb761 has also been shown to enhance cognition by increasing synaptic plasticity in the hippocampus [27]. Additionally, EGb761 has been proven to have cardiovascular protective effects in myocardial ischemia-reperfusion injury mediated through targeting NOS and NO production in the injured central nervous system[28]. In accordance with our present data, pretreatment with EGb761 has been found to attenuate up-regulation of cNOS and iNOS in the brain and have neuroprotective effects in hyperthermic brain injury[29]. A previous report has also shown that inhibition of the up-regulation of NO produced by iNOS reduced apoptosis in traumatic SCI models[30]. In the present study, our findings suggest that EGb761 has a neuroprotective effect on avulsion-induced motoneuron injury by significantly attenuating avulsion-induced up-regulation of cNOS and iNOS activities in the spinal cord and nNOS expressions in injured motoneurons. Thus, it is possible that down-regulation of iNOS activity by EGb761 treatment might be an effective therapy for root avulsion injury.

Previous studies have shown that all three of the NOS isoforms, nNOS, iNOS, and eNOS, are up-regulated in the injured spinal cord[31]. Our present data confirmed that cNOS activity in injured spinal segments was markedly increased, regardless of whether there was treatment with EGb761. The cNOS consists of both nNOS and eNOS. In the present study we did not specifically quantify eNOS activity in injured spinal segments; however, the endothelial cells of blood vessels in the ipsilateral ventral horns were intensely stained in the NADPH-d reaction, indicating an up-regulation of eNOS in the injured spinal cord. A consensus has been reached that eNOS acts as a neuroprotective agent during the central nervous system injury[32], as high expression of eNOS in the endothelial cells of blood vessels may increase blood flow and therefore aid in the survival of injured neurons[33,34]. However, there are still debates about the role of nNOS in CNS injury. Many previous studies have considered NO produced by the nNOS to be neuroprotective[8], while other studies have found NO produced by nNOS to be neurotoxic [35]. Kwak et al., found that nNOS was actually protective against cell death at early stages of the injury and was constantly expressed in neurons, yet aberrant neuronal expression of nNOS could result in the loss of its neuroprotective role [36]. We agree with the opinion that factors such as the concentration range of NO, the redox state of the molecule, the cell type source, and the environment in which the NO is produced by nNOS appear to determine the role of nNOS in the CNS[37]. Normally there is no nNOS labeling by immunohistochemistry in spinal ventral horn motoneurons[38], but labeling is apparent in neurons located in the dorsal horn and around the central canal [4,25]. Our present study further confirmed that avulsion induced an increase in nNOS activity in ipsilateral ventral horn motoneurons. It is possible that the increase in nNOS activity in the first 2 weeks might play a neuroprotective role in avulsion injury, a notion based on a number of observations. First, motoneuron loss was mild within the first 2 weeks following avulsion with or without EGb761 treatments. Second, our previous study showed that down-regulation of nNOS protein in injured ventral horn motoneurons augmented the subsequent motoneuron loss[15,22]. Finally, the present data further demonstrated a remarkable loss in motoneurons beginning around 4 weeks after avulsion, a decline which was correlated not only with increased iNOS activity but also with decreased cNOS activity in the spinal cord.

In summary, the present study showed that avulsion-induced motoneuron death was correlated with increased iNOS activity and changes in cNOS activity in the injured spinal cord, as well as nNOS expression in injured motoneurons. EGb761 treatment diminished avulsion-induced motoneuron death by attenuating the avulsion-induced NO production and cNOS, iNOS activities in the injured spinal cord and nNOS expression in injured motoneurons.

## Competing interests

The authors declare that they have no competing interests.

## Authors' contributions

The authors of this paper indicated in the title made substantial contributions to the following tasks of research: initial conception and design (XC, LFL, JZ, LLW, FLL, SL, LHZ); administrative, technical, or material support (XC, LFL, JZ, LLW, FLL, LHZ); acquisition of data (XC, LFL, JZ, LLW, FLL, SL, LHZ); laboratory analysis and interpretation of data (XC, LFL, JZ, LLW, FLL, SL, LHZ); drafting of the manuscript (XC, JZ, LHZ); critical revision of the manuscript for important intellectual content (XC, JZ, LLW, SL, LHZ). All authors read and approved the final manuscript. The views expressed herein are those of the authors and not necessarily their institutions or sources of support.
